# Potential and Challenges of a Targeted Membrane Pre-Fouling: Process Performance of Milk Protein Fractionation After the Application of a Transglutaminase Treatment of Casein Micelles

**DOI:** 10.3390/foods14152682

**Published:** 2025-07-30

**Authors:** Michael Reitmaier, Ulrich Kulozik, Petra Först

**Affiliations:** 1 Associate Professorship of Food Process Engineering, Department of Life Science Engineering, TUM School of Life Sciences, Technical University Munich, 85354 Freising, Germany; michael.reitmaier@tum.de; 2 Chair of Food and Bioprocess Engineering, Department of Life Science Engineering, TUM School of Life Sciences, Technical University of Munich, 85354 Freising, Germany; ulrich.kulozik@tum.de

**Keywords:** microfiltration, dynamic membrane, skim milk, micellar casein, milk protein fractionation, mem-brane fouling, transglutaminase

## Abstract

The covalent cross-linking of caseins by the enzyme transglutaminase (Tgase) stabilizes the structure of casein micelles. In our study, the effects of a pretreatment of skim milk (SM) by Tgase on milk protein fractionation by microfiltration were tested. Tgase was found to induce amount-dependent modifications of all milk proteins in SM and a reduction in deposit resistance for laboratory dead-end filtrations of up to 20%. This improvement in process performance could partially be confirmed in pilot-scale cross-flow filtrations of Tgase-pretreated SM and micellar casein solutions (MCC). These comparative trials with untreated retentates under a variation of ΔpTM (0.5–2 bar) at 10 and 50° revealed distinct differences in deposit behavior and achieved the reduction in deposit resistance in a range of 0–20%. The possibility of pre-fouling with enzymatically pretreated MCC prior to SM filtration was also investigated. Under different pre-fouling conditions, practical modes of retentate change, and pre-foulant compositions, a switch to untreated SM consistently resulted in an immediate and major increase in deposit resistance by 50–150%. This was partially related to the change in the ionic environment and the protein fraction. Nevertheless, our results underline the potential of Tgase pretreatment and pre-fouling approaches to alter filtration performance for different applications.

## 1. Introduction

The fractionation of milk proteins by cross-flow microfiltration (MF) from skim milk (SM) has become a standard unit operation in dairies to obtain micellar casein concentrate (MCC) and whey proteins in a nearly unmodified and purified state [1]. At ~10 or 50 °C, to diminish bacterial growth, membranes with a pore size of approximately 0.1 μm are utilized to retain caseins in a concentrated retentate phase, whereas whey proteins are transferred to the permeate/filtrate phase [2]. The filtrate flow rate and transmission of whey proteins are impeded by distinct deposit formation on the membrane surface due to an accumulation of milk proteins and pressure-related compaction. Although various approaches have been undertaken to reduce the additional deposit resistance (R_D_) provided by this fouling layer, this limitation has not yet been satisfactorily overcome [3]. Caseins are reported to be the major fouling constituents for MF of SM, even forming a gel-like fouling layer in direct proximity to the membrane [4,5]. They are mainly present in milk in the form of spherical assemblies called casein micelles (CM), which show a size distribution of approximately 20–400 nm and consist of the monomers κ-Casein, α_S2_-Casein, α_S1_-Casein, und β-Casein in a ratio of 1.3:1:4:4 [6]. While protein interactions and colloidal calcium phosphate stabilize the spherical structure, colloidal stability against aggregation of CM is provided by the hydrophilic part of k-casein present at the micellar surface [7]. The open-pore architecture of CM enables a continuous exchange with the surrounding serum phase, leading to structural and compositional changes in response to various chemical and physical alterations [8]. During industrial milk protein fractionation by cross-flow MF, a diafiltration (DF) step is typically applied wherein deionized water is added to wash out further whey proteins, lactose, and minerals, thereby increasing the purity of the casein fraction [1]. In the course of DF, a continuous increase in flux related to a reduced deposit resistance has been reported for MF of SM [9,10]. Besides some positive effects of a viscosity reduction in the milk serum phase, this is mainly related to changes in the deposited casein fraction induced by the alteration in the ionic environment, predominantly the reduction in the calcium content [9,11].

Caseins are described to be an excellent substrate for polymerization by the formation of isopeptide bonds between glutamine and lysine residues by the enzyme transglutaminase (Tgase) [12]. In milk, this covalent cross-linking is reported to occur exclusively intramicellar, converting casein micelles from association colloids to nanogel particles with increased stability against externally induced disintegration [12,13,14,15,16]. The effect of applying Tgase in MCC prior to MF at 5 °C was studied by Puri et al. [17]. These authors observed a significant reduction in the transmission of β-casein monomers, which typically reduces the purity of whey proteins in permeates obtained from MF at ~10 °C. The reported data also indicate a possible reduction in R_D_ related to the Tgase pretreatment. This was attributed to a reduced availability of reactive side chains at the surface of CM and/or a potential fouling contribution of β-casein in the untreated feed. Vasbinder et al. [18] assumed that parts of the CM are less flexible due to cross-linking by Tgase. Increasing the transmembrane pressure (Δp_TM_) as the driving force in MF could theoretically provide higher filtrate flow levels. However, for milk systems, this induces elongation and compression of deposited casein micelles [5,19], resulting in an increase in R_D_, which counteracts the increase in flux and reduces whey protein transmission into the permeate [20]. We hypothesize that deposit layers built from Tgase-pretreated casein micelles might be less compactable due to structural reinforcement by covalent binding. Thus, we believe that with a pretreatment of CM involved in deposit formation, a possible increase in process performance could be achieved both at 10 and 50 °C, especially at Δp_TM_ levels higher than 0.2 bar, as applied by Puri et al. [17]. A benefit of the direct application of Tgase in SM prior to MF was a higher degree of final casein concentration [21] and further applicability of the treated casein fraction for improving the structure of different dairy products [14]. Our first aim was to clarify to what extent Tgase pretreatment of SM could increase process performance under different pressure conditions for filtration at both 10 and 50 °C. As an undesired side effect, whey proteins could also be modified in their structure and consequently not be derived in a native state. Although caseins are reported to be preferably cross-linked by Tgase in this mixed protein system [22], whey proteins could also be altered in isolated form by large amounts of Tgase [23]. Unlike Hinz et al. [22], who observed no cross-linking of whey proteins in SM treated with Tgase, other studies indicate a small extent of cross-linking of whey protein besides caseins in different milk protein substrates [24,25]. Within the frame of our trials, alterations in the denaturation degree of whey proteins in differently Tgase-treated SM samples were therefore evaluated.

In our view, the application of Tgase could be a promising option to stabilize a casein fouling layer in an optimized state against a subsequent change in the retentate composition. Building a deposit from particles other than the actual feed material has already been suggested as a so-called ‘dynamic membrane’ for the improvement in various wastewater filtration processes [26]. As the applied shear limits deposit growth in cross-flow conditions, a simple additional effect on R_D_ for different feeds filtered in series could be avoided [27]. To our knowledge, targeted pre-fouling for milk filtration has only been tested by Obermeyer et al. [28], who described an improved retention behavior of ultrafiltration and reverse osmosis processes after targeted pre-fouling with milk proteins.

The related investigations were carried out in the following experimental setup: Firstly, filtration tests were conducted with the aim of investigating the impact of different Tgase pretreatments of SM on laboratory dead-end MF applying different Δp_TM_. Secondly, in cross-flow MF trials applying pressure cycling, the transferability of effects achieved by a targeted Tgase treatment for SM and MCC was tested. Finaly, single cross-flow filtration tests were conducted with the aim of transferring effects on R_D_ achieved by pretreated MCC to follow up filtrations of untreated SM.

## 2. Materials and Methods

### 2.1. Retentate Material

Short-time pasteurized SM (73 °C, 20 s) was supplied by a local dairy (Molkerei Weihenstephan GmbH & Co. KG, Freising, Germany) in a chilled state and stored for a maximum of four days at 4 °C before use in laboratory- and pilot-scale filtrations. Micellar casein (GermanMicell MCC 85 premium, Sachsenmilch Leppersdorf GmbH, Wachau OT Leppersdorf, Germany) and milk protein isolate (MPI) powder (TMP 85, MILEI GmbH, Leitkirchen im Allgäu, Germany) were also used to prepare further casein-containing feed material for pilot-scale experiments. Simulated milk ultrafiltrate containing lactose and the major salts of the milk serum phase (SMUF+L) was prepared from deionized water, chemical salts, and lactose monohydrate (Sigma-Aldrich; St. Louis, MO, USA) as described by Dumpler et al. [29]. Solutions of MCC or MPI containing 2.8% casein were prepared by premixing appropriate quantities of powder with preheated deionized water or SMUF+L in a shear pump (FSP 712/124, FRISTAM Pumpen KG (GmbH & Co.), Hamburg, Germany) at 50 °C for 15 min, followed by valve homogenization at 500 bar and 50 °C (HPH Rannie 56 type 16.56H, APV Gaulin GmbH, Lübeck, Germany) to obtain solutions of fully reconstituted CM. As described by Warncke and Kulozik [30], only particles in the size range of casein micelles were detectable by laser diffraction analyses (Malvern Mastersizer 2000 + Malvern Hydro 2000S, Malvern Instruments GmbH, Herrenberg, Germany) of the derived solutions.

### 2.2. Compositional Analysis of Filtration Media

RP-HPLC according to Dumpler et al. [31] was carried out to quantify casein monomers and major whey proteins in the retentate media and alterations in their native concentration induced by TGase in SM. The overall protein content of the retentate media and permeates of SM was quantified according to Dumas (Vario MAX cube, Elementar Analysensysteme GmbH, Hanau, Germany) using a conversion factor of 6.38. Calcium concentrations of SM and MCC were assessed by flame photometry (Elex 6361, Eppendorf AG, Hamburg, Germany).

### 2.3. Enzymatic Pretreatment with Transglutaminase (Tgase)

Tgase (Activa^®^ YG, Ajinomoto Foods Europe, Paris, France) with an activity of 100 U/gram was added in amounts of 0, 1, 2, or 3 units per g protein (U/g_protein_) to the different preheated feed solutions under stirring at 50 °C for 3 min. After an incubation time of 30 min at this temperature, the samples were batch pasteurized at 63 °C for 30 min to inactivate Tgase and cooled below 10 °C [32]. For this heat treatment and short-time pasteurization of SM, a relatively low denaturation degree of the whey protein fraction of up to 15–25% has been described [33,34]. This mild heat treatment of SM is also industrially applied to preserve a proper microbiological state during MF and, at the same time, alter the structure of CM and its behavior in filtration only to a very limited degree [1,35,36]. For lab-scale experiments, heating was carried out in a glass beaker with an overhead stirrer, two separate water baths, and ice water. For pilot-scale filtrations, a double-walled vessel connected to a tubular heating system and equipped with a stirrer to quickly heat and cool 31 kg of feed material was used. Feed materials were stored overnight at 4 °C before use in filtration tests.

The effect of Tgase pretreatment on individual milk proteins in SM was evaluated by sodium dodecyl sulphate polyacrylamide gel electrophoresis (SDS-PAGE). Detection under UV light was carried out after protein separation on a Mini-PROTEAN^®^ TGX™ gradient gel (4–20%, Bio-Rad Laboratories, Inc., Hercules, CA, USA) under non-reducing conditions at 300 V for 35 min with milk protein standards (Sigma-Aldrich; St. Louis, MO, USA). RP-HPLC was then also carried out to quantify the difference in the content of native casein monomers and whey proteins induced by the different Tgase treatments.

### 2.4. Dead-End Filtrations on a Laboratory Scale

Dead-end filtration experiments at 20 °C were conducted using an Amicon 8050 frontal filtration cell (Millipore Corp., Billerica, MA, USA) equipped with single-use 0.1 µm MF membranes (PVDF, 44 mm, Durapore^®^, Merck Millipore Ltd., Tullagreen, Irland). Dynamic pressurization was carried out to adjust Δp_TM_ with nitrogen using a digital electronic pressure control unit (AL-PRESS, Bronkhorst, Ruurlo, NL, USA). The permeate mass was weighed and recorded every 5 s during filtration on a precision balance (LA 1200 S, Sartorius AG, Göttingen, Germany) using Sartocollect Light software (Version 1.0, Sartorius AG, Göttingen, Germany). Applying Equation (1), the mass-based flux (*J*) can be calculated from the measured weight gain (*dm*) per time interval (*dt*) related to the membrane surface area (A) 1.52 × 10^−3^ m^2^,

(1) $$J=\frac{1}{A}\cdot \frac{dm}{dt}$$

As described in detail by Reitmaier et al. [9], the dry weight (*m_m_*), water flux (*J_w_*) during pre-washing at a *Δp_TM_* of 0.7 bar, weight when wetted with deionized water (*m_w_*) to account for filled permeate channels, and membrane resistance (R_m_) according to Equation (2) were determined for every membrane before SM filtration.

(2) $$R_{M}=\frac{{\Delta p}_{TM\;}}{\eta_{w\;}J_{w}}$$

One of the differently pretreated SM samples (m = 10 g, ϑ = 20 °C) was then placed on top of the membrane. A total amount of 2 g of permeate was filtered through the membrane at 0.5 or 3.0 bar Δp_TM_. The resistance of the deposit layer (*R_D_*) was calculated according to Equation (3) from the final flux (*J*) and *R_M_*. A viscosity of η = 1.17 × 10^−3^ Pas was considered for the milk serum as the actual permeate phase [37].

(3) $$R_{D}=\frac{{\Delta p}_{TM\;}}{\eta_{per}\;J}-R_{m}$$

After slow pressure release, excess milk was thoroughly poured off, and the mass after filtration (*m_F_*) could be gravimetrically obtained to calculate the deposited mass (*m_D_*) using Equation (4).

(4) $$m_{D}=\;m_{F}-m_{w}$$

The dry matter of the fouled membrane (DM_D+M_) was analyzed (CEM Smart Turbo, CEM-GmbH, Kamp-Lintfort, Germany) to derive the dry mass within the deposited mass (*DM_D_*) using Equation (5).

(5) $${DM}_{D}={DM}_{D+M}\;\times \;m_{D}-m_{m}$$

### 2.5. Cross-Flow Filtration Tests on a Pilot Scale

An amount of 30 kg of the differently pretreated feed materials was briefly kept in lukewarm water to reach 10 °C or hand-stirred and heated in a water bath (53 ± 1 °C) to 50 °C over 15 min. Filtrations were conducted on a pre-tempered filtration rig (SIMA-tec GmbH, Schwalmtal, Germany) described in detail by Schiffer et al. [20], equipped with a ceramic multi-channel membrane (0.1 µm, Type 19/3.3, A = 0.24 m^2^, L = 1.020 m, atech innovations GmbH, Gladbeck, Germany). The cleaning of the membrane was carried out according to Schiffer et al. [20], comprising an alkaline and acidic step followed by alkaline pretreatment before final rinsing with deionized water. After washing out the purified water from the filtration rig, 25 L of feed material was filtered in a ‘constant-volume’ batch mode [38] to assess the fouling behavior under recirculation of retentate and permeate to the feed tank during pressure variation tests. Pressure cycling was carried out with a step-wise increase in Δp_TM_ followed by a decrease in steps of 0.5 bar for at least 30 min. This enabled the evaluation of pressure- and time-dependent aging behavior of a fouling layer built from retentates with unchanged composition in a single filtration run [20]. Δp_TM_ levels of typically applied 0.5–2 bar were adjusted in series by regulating the pump speed and throttle valves positioned before and after the membrane housing, as described by Schiffer et al. [20]. This pressure variation induces a gradual deposit compaction and inhibition of whey protein transmission as well as permeate flow up to a constant level at the so-called ‘limiting transmembrane pressure’ [20,39]. A constant wall shear stress (τ_w_) of 115 Pa was applied for all settings to ensure a comparable cross-flow regime along the membrane, leading to efficient fouling reduction for milk filtration [40]. Only during the diafiltration experiment permeate was continuously led off the filtration rig and replaced by a manual supply with SMUF+L, maintaining the fill level constant. Three washing cycles with SMUF+L, each equaling the retentate volume of 25 L (3-step diafiltration), led to a nearly complete change in the permeate phase of ~95% [9]. For all experiments, J over filtration progress was derived using Equation (1) from gravimetric measurements of the permeate flow. To further assess R_D_ during filtrations with the different feeds containing casein using Equation (3), R_M_ was derived from the initially measured pure water flux using Equation (2). During pilot-scale experiments, the concentration of native whey proteins was determined in permeates (c_WP,Per_) and retentates (c_WP,Ret_) to calculate their relative transmission (P_WP_) into the permeate. After precipitation of caseins and denatured whey proteins at pH 4.6 with 0.1 M HCl, samples were filtered through a 0.45 µm syringe filter. The protein content of the derived solutions is nearly completely related to native whey proteins [41,42]. It was quantified according to Dumas (Vario MAX cube, Elementar Analysensysteme GmbH, Hanau, Germany) using a conversion factor of 6.38 and applied in Equation (6).

(6) $$P_{WP}=\frac{c_{WP,Per}}{c_{WP,Ret}}$$

### 2.6. Data Analysis

Data were plotted with Origin 2023 (OriginLab Corporation, Northampton, MA, USA). For data obtained from two completely separate preparative and technical replicate filtration trials (*n* = 2), error bars depict the actual values with the derived mean.

## 3. Results

### 3.1. Composition of Filtration Media and Analyses of TGase-Induced Modifications in SM

The milk protein compositions of the different feed solutions in unmodified conditions were assessed by RP-HPLC, and the results are provided in Table 1.

While the casein ratio was comparable for all substrates, the whey protein content of MCC was markedly reduced compared to SM and MPI. The overall protein content was also at a reduced level of 2.94 ± 0.16 mg ml^−1^ in MCC compared to 3.68 ± 0.13 mg ml^−1^ in SM and 3.61 mg ml^−1^ in MPI. For the main feed materials SM-0U and MCC-0U, total calcium concentrations of 1533 ± 36 and 896 ± 62 mgL^−1^, respectively, were detected. The lower content of MCC-0U is related to diafiltration with deionized water during industrial production, leading to a washout of serum minerals.

**Figure 1:**
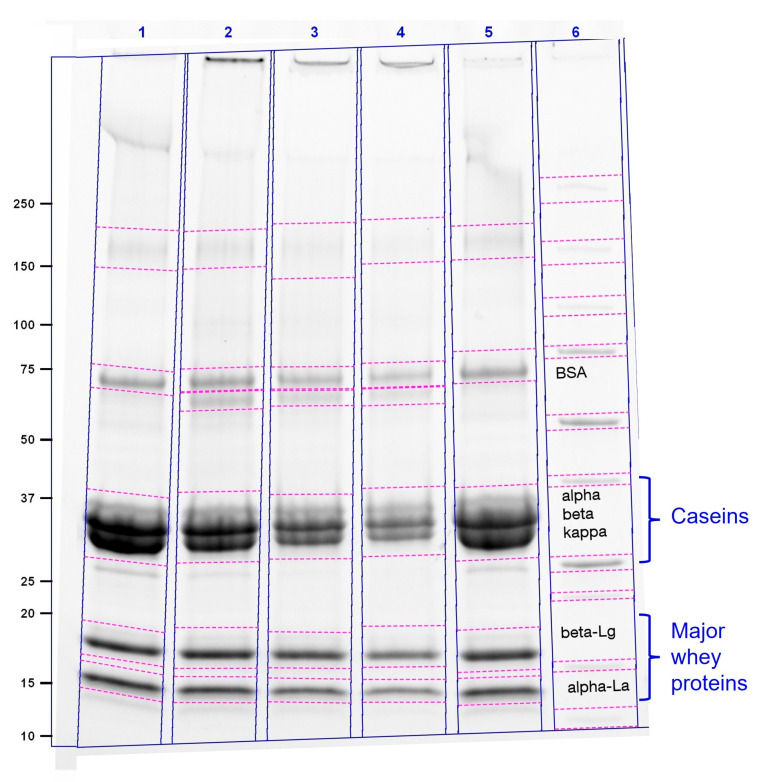
SDS-PAGE electrophoretogram of skim milk (SM) samples treated with different amounts of Tgase and heated for inactivation; 1: SM with 0 U/g_protein_, 2: SM with 1U/g_protein_, 3: SM with 2U/g_protein_, 4: SM with 3U/g_protein_, 5: SM not inactivation heated as a reference, 6: milk protein standards.

Different Tgase treatments of SM prior to MF trials under variations in the added enzyme amount were carried out. Based on data from the literature related to changes in caseins in SM with the chosen Tgase substrate for other applications [43,44,45], treatments with enzyme amounts of 0–3 U/g were selected. As CMs are reported to be depleted in β-casein and more highly hydrated in milk after cooling, the enzymatic reaction was carried out after heating to 50 °C to achieve cross-linking of CM at a nearly reset natural state [46]. To maintain the cross-linking effects achieved after 30 min at a defined level during further application, heating to 63 °C for 30 min was applied as an enzyme inactivation step while preventing major whey protein denaturation [32]. Due to inconsistent information from the literature on changes in whey proteins induced by Tgase treatment of milk, changes in the native state of all major milk proteins were analyzed. SDS-Page results showed that with increasing amounts of added Tgase, the proportions of native casein monomers and major whey proteins were progressively reduced (Figure 1). The inclusion of an SM sample heated for 30 min at 63 °C without the addition of Tgase showed that the effect of the heat treatment applied for enzyme inactivation was negligible.

These observations indicate structural changes in caseins due to the desired cross-linking within CMs but also modifications of whey proteins as an undesired side effect. CMs modified with Tgase in MCC isolated by MF or in milk protein concentrate/isolate obtained by ultrafiltration could indeed be used for structural optimization of yoghurt or ice cream [47,48]. Due to the observed structural changes in whey proteins, MF of Tgase-pretreated SM is not an appropriate way to obtain a maximum of their native form. As denatured whey proteins are reported to be selectively retained during MF [49], improvements in the overall process performance through the pretreatment of SM are nevertheless possible. As the peaks of casein monomers and whey proteins overlay in size exclusion chromatography [50], RP-HPLC was tested to provide quantitative information on the portion of single major milk proteins modified by Tgase. A decrease in the amount of native casein monomers following the amount of Tgase added to SM was also apparent in these analyses (Table 2). The observed change in the relative nativity of single caseins following the change in the typically reported order of κ >β > α_s1/s2_ can be attributed to their differences in spatial accessibility within CMs [13]. The peak areas normally attributed to whey proteins were found to be increased for the Tgase-treated samples. As this is clearly related to a methodical error when applied to this type of sample, quantities could not be specified. This effect was confirmed through analyses using another RP-HPLC method according to Heidebrecht et al. [51], where peak areas of different whey proteins were found to be increased or decreased after treatment with Tgase.

The observed rise is most likely related to an overlapping of the whey-protein-specific peak areas with peaks of cross-linked proteins, which has, to our knowledge, not been reported so far. Consequently, this analytical approach could not further be considered for the attended appropriate quantification of Tgase-induced changes in the content of native whey proteins. However, the comparison of the amount of native whey proteins as major target compounds in the obtained permeates was crucial for the desired assessment of different filtration experiments. Therefore, a quantification of proteins according to Dumas after a precipitation step at pH 4.6 was applied for the evaluation of the transmission of native whey proteins in our pilot-scale experiments. Future investigations combining methods like LC-MS or native PAGE could address characterization and quantification of Tgase-induced polymers which interfere in RP-HPLC.

### 3.2. Testing of Different Tgase Treatments in Laboratory-Scale Dead-End Filtration

The flux measurements for dead-end filtrations of SM treated with 0–3 U/g Tgase revealed a 10–20% reduction in R_D_ for 2 and 3 U/g Tgase at 0.5 bar as well as 3 bar Δp_TM_ (Table 2). Due to the found maximum effect of Tgase on the deposit resistance at 2 U/g indicating a saturation effect, this amount was chosen for all further Tgase treatments prior to filtration trials. In our further analysis of the deposits formed from the differently pretreated SM, no deviations in the deposited mass or its water content respectively dry matter were observed (Table 3).

For trials in a comparable filtration setup, it was found that when varying the calcium content or pH in the milk serum surrounding the CM, both the R_D_ and water content of the deposit layers significantly changed [9,52]. These parallel alterations were attributed to differences in interactions, composition, and colloidal properties of the CM caused by the applied changes of the ionic environment. Tgase treatment of CM has been described to result in decreased repulsion due to covalent fixation of k-casein at the surface [13] and increased hydrophobicity [53]. These differences in the interaction of CM could have potentially also resulted in alterations in the hydration of deposit layers. However, the comparable mass and DM data show that the observed differences in R_D_ induced by the Tgase pretreatment are rather related to a reduction in the physical compression reaction towards the applied pressure, most likely due to their reduced structural flexibility [18].

### 3.3. Cross-Flow Microfiltration of Untreated and Tgase-Pretreated Skim Milk

**Figure 2:**
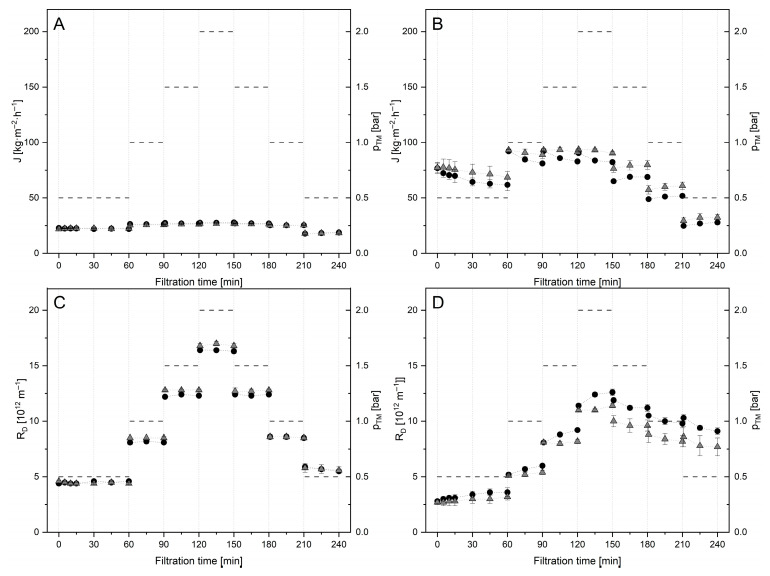
Flux (J) and deposit resistance (R_D_) obtained during filtrations of skim milk without (SM-0U, ●) and with Tgase pretreatment (SM-2U, 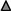) at 10 °C (**A**,**C**) and 50 °C (**B**,**D**), respectively, under sequential step-wise adjustment of Δp_TM_ (-).

During pilot-scale filtration experiments of untreated (SM-0U) and Tgase-pretreated (SM-2U) SM, differences in J and R_D_ at 10 °C and 50 °C were observed (Figure 2).

For all filtrations, the increase in Δp_TM_ resulted only in a slight rise in flux. This is attributed to the deposit-dominated filtration typically occurring in SM filtration [20,54,55]. In accordance with the observations of Hartinger et al. [54] for polymeric membranes, J and R_D_ did not change over the holding time at all applied pressure levels at 10 °C (Figure 2A,C). This indicates that no relevant rearrangements within the deposited mass take place at this temperature over filtration duration at constant Δp_TM_. This might be related to an immediate and dominant gel-like state of the deposit structure as an increased voluminosity of casein micelles at this temperature fosters their gelation in a concentrated state at the membrane surface and reduces their compressibility [56,57,58]. In contrast, for filtrations at 50 °C, we observed a slight decrease in J and an increase in R_D_ over different filtration times for each level of Δp_TM_ during its step-wise elevation (Figure 2B,D). This so-called aging of the deposit layer that could be related to a continuous specific accumulation of denatured whey proteins over filtration progress was also reported by Hartinger et al. [54] and Subhir et al. [59]. Except at 0.5 bar, similar levels of J and R_D_ were achieved for single pressure settings during an increase from 0.5 to 2 Δp_TM_ and a subsequent decrease to 0.5 Δp_TM_ at 10 °C. However, the values of J and R_D_ obtained at 50 °C were found to be at lower and higher levels, respectively, during step-wise pressure decrease when compared to the corresponding pressure settings during its increase. This is in accordance with earlier reports on SM filtrations at 50 °C using polymeric or ceramic membranes, attributing this to pressure rather than time effects [54,60]. During pressure increase, a critical condition will be attained that leads to partially irreversible alterations in the deposit layer. This is described to arise from deposit compression, related deformation, and compaction of CM as well as cross-linking of proteins involved in the deposit, leading to intensified gelation [40,54,60]. Besides a possible contribution of reactive thiol sites of whey proteins at 50 °C, caseins could possibly undergo irreversible gelation at this elevated temperature in a highly concentrated state. Thomar and Nicolai [61] showed that the critical temperature that leads to heat-induced irreversible gelation involving hydrogen bonding, calcium, and hydrophobic interactions reduces with an increasing concentration of CM. Their study was limited to a maximum casein concentration of 160 g l^−1^ requiring 90 °C to induce irreversible gelation at the typical milk pH of 6.7. However, the much higher concentration of approximately 400 g l^−1^ at the membrane surface, as reported by Doudiès et al. [40], could markedly reduce the critical temperature. In contrast, the ‘cold gelation’ of CM when concentrated at low temperatures due to overlapping of their hairy layer and particle jamming is reported to be reversible [58,62]. This temperature-dependent type of interaction leading to the gelation of CM in a concentrated state could account for the different behaviors of the deposit layers observed at 10 and 50 °C when reducing Δp_TM_. During pressure release at 50 °C, for each setting of Δp_TM_, a slight increase in J over different filtration durations was observed, meaning there is at least a partial reversibility of the pressure-induced decrease in R_D_ over time, which can be related to physical swelling [40]. At 10 °C, no impact or a slight negative impact on flux was observed for filtrations of SM-2U compared to SM-0U. R_D_ could have been slightly increased due to a Tgase-induced reduction in the voluminosity. This can be ascribed to a loss of casein mobility in SM-2U, which hinders the temperature-dependent transition of β-casein between the micellar and monomeric stages and the structural swelling of CM upon cooling, known to occur in untreated SM [16,57].

For filtration at 50 °C, an increase in J was achieved by the applied Tgase treatment at all adjusted settings of Δp_TM_ (Figure 2B). For SM-2U, R_D_ was found to be at lower levels, and the difference increased with the enhancement in Δp_TM_. Furthermore, the time-dependent increase at single pressure settings was reduced for Δp_TM_ ≥ 1.0 bar and a major benefit towards SM-0U emerged during step-wise pressure release. This means that the Tgase treatment could partially overcome the compaction of deposits by Δp_TM_ and deposit aging over time at 50 °C, most likely due the hypothesized reduced compressibility of deposited CM. This effect was not achieved at 10 °C due to the already reduced compressibility of CM in SM-0U at a low temperature, which also confirms our main hypothesis.

**Figure 3:**
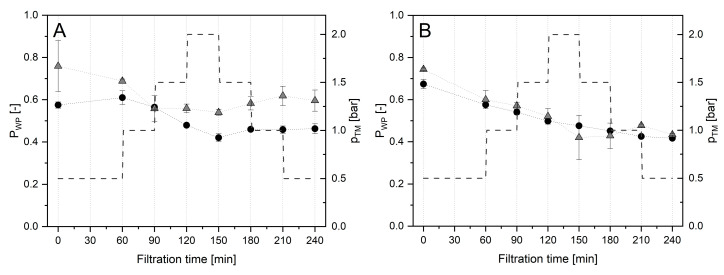
Transmission of native whey proteins (P_WP_) for SM-0U (●) and SM-2U (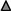) at 10 °C (**A**) and 50 °C (**B**) applying sequential step-wise adjustment of Δp_TM_.

The relative transmission of native whey proteins into the permeate was found to be slightly increased for SM-2U for filtrations at 10 °C (Figure 3A). At 50 °C, no difference for SM-2U compared to SM-0U was observed (Figure 3B). These results indicate that Tgase pretreatment of CM might not lead to reduced fractionation efficiency when used for pre-fouling prior to MF of untreated SM.

### 3.4. Cross-Flow Microfiltration of Untreated and Tgase-Pretreated Micellar Casein

**Figure 4:**
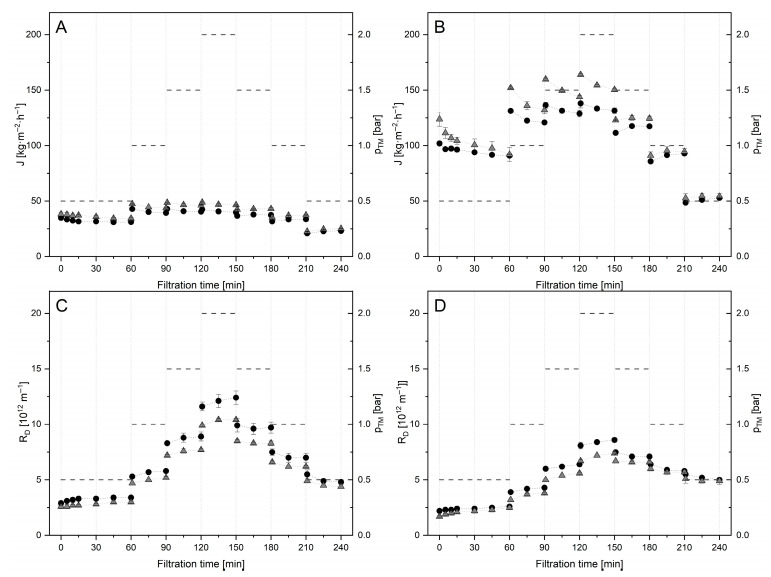
Flux (J) and deposit resistance (R_D_) obtained during filtrations of micellar casein without (MCC-0U, ●) and with Tgase pretreatment (MCC-2U, 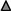) at 10 °C (**A**,**C**) and 50 °C (**B**,**D**) under sequential step-wise adjustment of Δp_TM_ (-).

The observed modification of whey proteins will cause a major hurdle for the treatment of SM with Tgase prior to industrial MF, as the recovery of whey proteins in a native state is one of the key benefits of this process. Tgase pretreatment could, however, be considered for achieving benefits in filtration performance for later application of the modified casein fraction in dairy products. However, the treatment of isolated casein micelles in MCC after the separation of native whey proteins seems to be a more promising option to gain potential benefits as a pre-fouling layer. Therefore, Tgase treatment of MCC (MCC-2U) was tested to overcome this issue and gain further advantages in filtration performance that could lead to a potential significant reduction in R_D_ in a subsequent MF of untreated SM. During filtration tests under a step-wise variation in Δp_TM_ with MCC-2U and untreated MCC (MCC-0U) at 10 °C and 50 °C, J and R_D_ (Figure 4) showed pressure-dependent behavior, deviating from the observations for SM (Figure 2).

During pressure increase, higher J and lower R_D_ values, respectively, prevailed for MCC-2U compared to filtrations of MCC-0U at both temperatures. As also observed for SM-2U at 50 °C (Figure 2D), the benefit of the Tgase-treated MCC regarding the reduction in R_D_ increased with higher pressure levels at 10 and 50 °C (Figure 4C,D). Interestingly, in contrast to the observations for SM-2U at 50 °C, the gap between R_D_ of MCC-2U and MCC-0U narrowed at 10 °C and was even lost at 50 °C during pressure release, and aging behavior over holding times was observed for all pressure levels. This indicates that the differences in the change of RD of Tgase-treated SM compared to untreated SM can not only be related to modifications of the physical compressibility of CM within the formed deposits. As MCC is reduced in WP and ions, interactions with these compounds are likely to have also been modified in SM-2U leading to reduced RD. While MCC (Figure 4B,D) showed a similar pressure- and time-dependent filtration behavior as SM (Figure 2B,D) at 50 °C, the observations for MCC at 10 °C (Figure 4A,C) are surprisingly in complete contrast to findings for SM at 10 °C (Figure 2A,C). For SM, no aging emerged, which the literature attributes to the increased voluminosity of CM at 10 °C. As CMs are described to have an even higher voluminosity in MCC than in SM, our findings counteract this explanation. Although the casein content was slightly lower in the MCC feed, we consider the differences in the behavior observed for SM and MCC to be related to a change in the interactions of CM with calcium ions and/or whey proteins in the formed deposits. The higher availability of calcium ions in the serum phase of SM is described to foster the cold gelation of caseins and whey proteins [63,64], as well as their potential interaction [65,66]. This might have led to the deviant observations for R_D_ in SM at 10 °C compared to SM at 50 °C and MCC at both temperatures. The increased levels of calcium, whey proteins, and osmotic pressure in SM could have resulted in a gelled state of parts of the deposit already at low Δp_TM_ values, as described by Qu et al. [5] for CM. Due to the reduced interaction with calcium/whey proteins in MCC, the effect of covalent stabilization of Tgase during step-wise pressure release was also reduced compared to SM at 50 °C. For SM-0U, a higher degree of interaction was most likely induced during pressure increase and holding times (aging), which was prevented by covalent stabilization of casein micelles in SM-2U. These results clearly demonstrate composition-dependent differences for the filtration of SM and MCC, underlining legitimate issues regarding a direct comparability of observations for one of these materials, as recently raised by Doudiès et al. [40].

**Figure 5:**
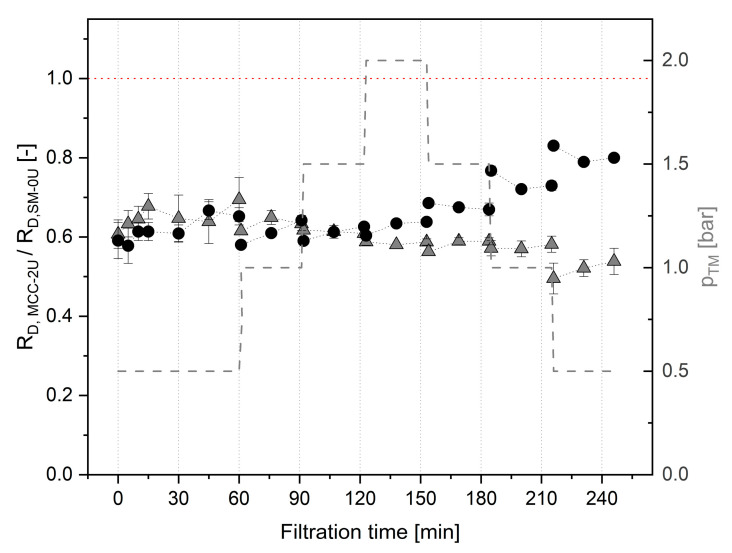
Relative deposit resistance (R_D,MCC-2U_/R_D,SM-0U_) obtained during filtrations of Tgase-pretreated micellar casein (MCC-2U) under sequential step-wise adjustment of Δp_TM_ (-) in comparison to filtrations of untreated skim milk (SM-0U) at 10 °C (●) and 50 °C (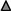).

One outcome of these tests with major relevance for our further investigations was that, compared to filtrations of SM-0U, a lower R_D_ value prevailed during the pressure ramp filtrations with MCC-2U at all pressure levels, both at 10 and 50 °C (Figure 5).

For filtration at 50 °C, a reduction in R_D_ in the range of 30 to 50% was observed over all pressure settings. At 10 °C, a similar reduction was achieved using MCC-2U compared to SM-0U during pressure increase. However, during step-wise pressure release, the advantage was reduced to 20% at 0.5 bar after 30 min. This is related to the deviant deposit behavior of SM at 10 °C and the aging of MCC deposits at 10 °C, both of which were observed independently of the Tgase treatment. In MCC-2U, a slightly lower level of the major foulant casein was applied, which might have partially contributed to the reduced R_D_ compared to SM-0U. The reduction can further be related to the swollen state of CM, calcium reduction in MCC [9,57], and lowering by Tgase pretreatment.

### 3.5. Tests to Implement the Setup of an Optimized Deposit Layer of MCC-2U Prior to MF of SM-0U

Different practical approaches to convey the observed reduction in R_D_ for filtrations of MCC-2U into targeted pre-fouling for a subsequent filtration of SM-0U were tested. MCC-2U retentates were directly replaced by SM-0U after finishing the pressure ramp testing depicted in Figure 5 comprising relatively strong physical deposit compression. For 10 and 50 °C filtration series, an immediate and considerable drop in J was observed when carrying out a subsequent filtration with SM-0U at a Δp_TM_ of 0.5 bar.

**Figure 6:**
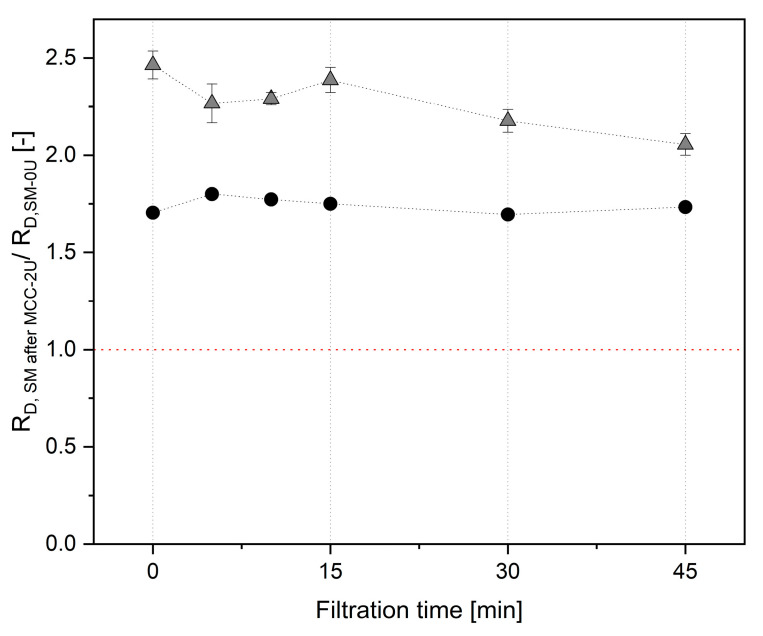
Relative deposit resistance (R_D, SM after MCC-2U_/R_D,SM-0U_) obtained during filtrations of untreated skim milk (SM-0U) at 0.5 bar Δp_TM_ following pre-fouling with Tgase-pretreated micellar casein (MCC-2U) under sequential step-wise adjustment of Δp_TM_ in comparison to filtrations of SM-0U with clean membrane at 10 °C (●) and 50 °C (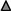) and 0.5 bar Δp_TM_.

As shown in Figure 6, there was also a major increase in R_D_ to a considerably higher level compared to reference SM-0U filtrations at Δp_TM_ = 0.5 bar using a clean membrane. This effect was found to be independent of the practical realization of the retentate phase change: at 50 °C, pausing of filtration by stopping the pump and draining off MCC-2U, followed by refilling with 30 kg of pre-heated SM-0U and pressure readjustment, was tested. Although in this approach, the mixed phase is minimized, an additional effect of the short-time deposit decompression induced by the pressure drop was conceivable. Therefore, at 10 and 50 °C, a direct wash-out of the retentate by replacing MCC that was leaving the system over the retentate tube was additionally applied. A total of 65 L of pre-heated SM was used to flush the filtration system at a Δp_TM_ of 0.5 bar until switching back into recirculation mode at the standard retentate volume of 25 l. The results for 50 °C were comparable for both approaches and are depicted as one data set in Figure 6, with the standard error of the mean indicating the absolute differences for *n* = 2. This shows that the physical impact by deposit relaxation when completely pausing the process in the first approach did not influence the observed major increase in R_D_ when changing from MCC-2U to SM-0U. A reduced transmission of native whey proteins of P_WP_ = 0.54 at 10 °C and P_WP_ = 0.52 ± 0.07 at 50 °C, respectively, was observed after 45 min compared to filtrations of SM-0U on a clean membrane at 0.5 bar. This indicates, besides a major loss in filtration efficiency, a decrease in separation effectiveness when applying the pre-fouling approach that was initially carried out.

The increased Δp_TM_ of 2.0 bar applied in the first pre-fouling attempt could have led to a relatively intensive and irreversible fixation of the built deposit layer, which was originally intended to persist in changes induced by a change in SM-0U. However, this might have led to a strongly compressed and gelled state, impeding a potentially beneficial effect of MCC-2U as a pre-fouling material with reduced R_D_. Besides physical aspects of the retentate change, alterations related to differences in components in SM-0U compared to MCC-2U must be considered as possible causes for the observed additive effect on R_D_:

- Although pressure conditions were kept stable, the higher viscosity of SM-0U, which is mainly related to the presence of lactose, induces a possibly relevant abrupt change in the flow profile at the membrane surface to less turbulent conditions [26].
- The increased amount of relatively small whey proteins in SM-0U could lead to the abrupt pore blocking of the less compacted, more porous pre-fouling layer.
- The structural state of casein micelles in the retentate changes from a slightly swollen state in MCC-2U to a nearly natural and more compact state in SM-0U [54], which might also lead to a pore-blocking effect.
- The ionic environment changes from the water-like state in MCC induced by DF to the serum phase of SM-0U. The higher calcium level of the latter medium has been described to increase interaction within deposits built from natural casein micelles, resulting in their compaction [25]. Despite the Tgase treatment, comparatively strong inter- or intramicellar interaction and compaction might be induced in a deposit built from less regularly structured CM in MCC when increasing the calcium level and ionic strength.

To shed light on possible ways to gain an advantage of the reduced R_D_ after pre-fouling and on the possible contribution of compositional factors, further single trials were carried out. To minimize possible physical impacts during the changing process, the second approach of a retentate washout was used during further pre-fouling tests.

**Figure 7:**
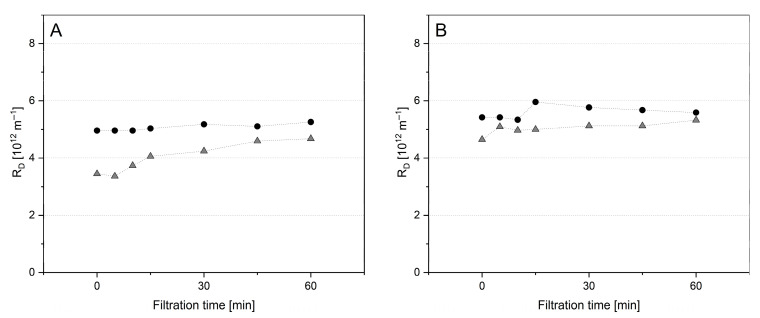
Deposit resistance (R_D_) obtained during filtrations of untreated skim milk (SM-0U) for pre-fouling (**A**) and subsequent filtration with a second SM-0U portion originating from the same source batch (**B**) at 10 °C (●) and 50 °C (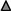) and at 0.5 bar Δp_TM_.

With the first set of single filtrations, an additive deposition of material when simply changing from SM-0U to a fresh portion of SM-0U originating from the same batch was tested (Figure 7).

Compared to the value obtained after 60 min of pre-fouling with SM-0U at 0.5 bar Δp_TM_ (Figure 7A), R_D_ was found to be at a relative level of 1.02 at 10 °C or 0.99 at 50 °C, respectively, when starting filtration with a fresh portion of SM-0U (Figure 7B). As this means a negligible change for both temperatures, the observed increase in R_D_ after pre-fouling with MCC-2U was not related to the changing process itself. These results also show that the additive accumulation of specific minor compounds in the deposit layer, as generally conceivable for the filtration of a new batch of milk on a pre-fouled membrane, can also be excluded.

**Figure 8:**
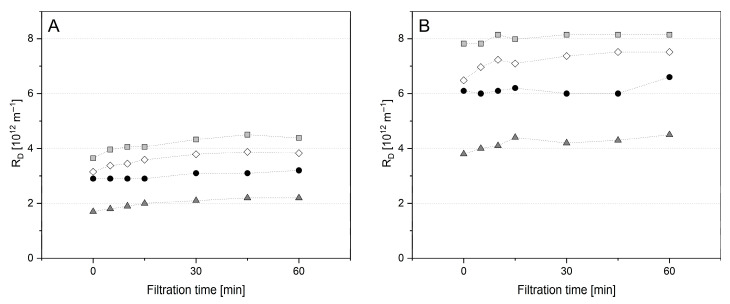
Deposit resistance (R_D_) obtained during pre-fouling filtrations (**A**) of each Tgase-pretreated micellar casein (MCC-2U, ●), milk protein isolate (MPI-2U, ⬦), micellar casein with lactose (MCC+L-2U, 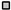) at 10 °C, and Tgase-pretreated micellar casein (MCC-2U, 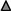) at 50 °C and related subsequent filtrations of untreated skim milk (SM-0U) (**B**) at 0.5 bar Δp_TM_.

During further pre-fouling tests with MCC-2U at 10 and 50 °C, a reduced Δp_TM_ value of 0.5 bar was applied over 60 min. Further retentates additionally tested at 50 °C were MCC adapted in viscosity to SM by the addition of lactose (MCC+L-2U) and milk protein isolate (MPI-2U) containing an amount of whey proteins comparable to SM-0U (Figure 8).

**Figure 9:**
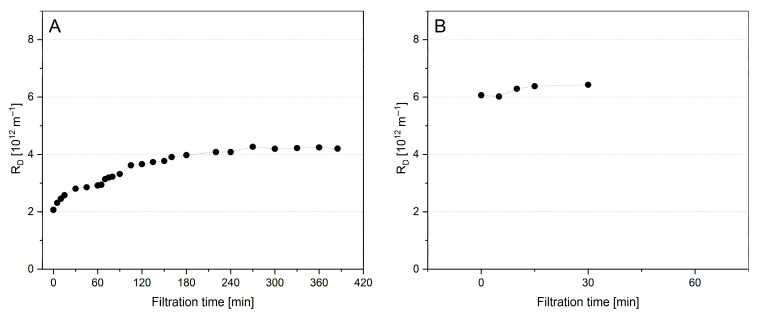
Deposit resistance (R_D_) obtained during diafiltration (**A**) of Tgase-pretreated micellar casein (MCC-2U, ●) with simulated milk ultrafiltrate containing lactose (SMUF+L) and related subsequent filtration of untreated skim milk (SM-0U) (**B**) at 50 °C and 0.5 bar Δp_TM_.

As already shown for the pressure ramp test in Figure 5, the R_D_ value for pre-fouling MCC-2U was higher at 10 °C than at 50 °C (Figure 8A). For MPI-2U, a higher R_D_ value was observed, which can be related to the additional amount of whey proteins. A further increased level was observed for MCC+L, which can be attributed to the higher retentate viscosity induced by lactose, resulting in modified flow profile [11]. For all of these pre-fouling tests, subsequent filtration with SM-0U also resulted in an abrupt and major increase in R_D_ (Figure 9B). Therefore, the observed major change in R_D_ occurred independently of the temperature, Δp_TM_, and could not be related to a change in retentate viscosity or whey protein content. Interestingly, the absolute levels of R_D_ achieved during the SM filtrations (Figure 8B) followed the order that had emerged in the prior pre-fouling step with different retentates (Figure 8A). The relative increase when switching to SM-0U was uniformly found on a level of ~100%, indicating direct dependence of R_D_ in SM filtration on the composition of the pre-built deposit layer. These results demonstrate, on the one hand, that fouling can be modified by pre-fouling depending on the composition of the retentate used. On the other hand, except for SM-0U, our tests all unfortunately resulted in a major additive effect instead of the hypothesized convoying of a reduction in R_D_. A final filtration experiment was conducted to additionally test the potential contribution of a change in the ionic environment and the role of casein micelles in their native state (Figure 9). A simple abrupt change in the ionic environment surrounding the serum phase of milk is hindered by the complexity of its salt system. To solely accomplish a change in the ionic environment with MCC+L-2U, the retentate was washed 3-fold with a priorly prepared SMUF+L. This change from the water-like composition of the serum phase to a milk-like ionic environment surrounding the CM resulted in a continuous increase in R_D_ (Figure 9A). This shows that the change to the ionic environment of milk is already related to a major increase in R_D_. Due to the high ionic strength and calcium availability, increased interaction and compaction of caseins and casein micelles are also likely to foster a deposit built from CM in water-like conditions [9,67]. However, a further strong increase in R_D_ when changing to SM-0U shows the substantial contribution of the addition of casein micelles and whey proteins in their native state. Due to their smaller size compared to the casein micelles originating from MCC or MPI, they could penetrate and block channels of the micelle network formed on the membrane surface during pre-fouling. In this way, a major increase in R_D_ has also been described for particle systems of mixed sizes by Fu and Dempsey [68] due to increased packing density. In combination with an abrupt change in the ionic environment when switching to SM-0U, this has likely resulted in the prompt formation of a highly compacted protein network, leading to the observed steep increase in R_D_.

Consequently, the applicability of potentially beneficial pre-fouling with MCC-2U was shown to be impeded by the change in the ionic environment and particle properties of the feed material. Alternative approaches to targeted pre-fouling could be examined when considering these newly discovered hurdles. The conceivable application of Tgase within a filtration device for the treatment of deposit layers formed under different conditions could result in deviant behavior. However, the effect of Tgase on casein micelles in an already formed compact deposit is less likely to emerge due to the reduced accessibility of reactive casein sites. Furthermore, the need for the inactivation of Tgase at elevated temperatures after application to avoid further uncontrolled reaction and labeling of obtained products renders this impractical.

## 4. Conclusions

Our investigations show that Tgase treatment of CM can be applied to reduce the membrane fouling of SM as well as MCC. Former studies on milk fouling during filtration have utilized either MCC or SM as a feed material. Our observations revealed distinctly different behavior of the formed fouling deposits, not only for Tgase-treated but also for untreated SM and MCC in response to pressure cycling at 10 and 50 °C. This underlines the role of interactions of different proteins and calcium in deposit aging and irreversibility following physical compaction during filtration. Therefore, our study sheds light on the effects of Tgase and on the composition-related causes of different observations when applying SM or MCC in MF, both containing CM but differing significantly in their further composition. A detected reduction in the native amount of whey proteins in SM renders the direct application of Tgase to enhance J in commercial milk protein fractionation impracticable. However, regarding a treatment to gain advantages with Tgase-modified CM in a concentrated state in dairy product applications, the results suggest performing filtration after enzymatic treatment. The applicability of Tgase-pretreated MCC to set up a fouling layer with significantly reduced R_D_ to induce a higher J value in a subsequent SM filtration process was impeded by changes in the protein particle system and the ionic environment. However, the results also demonstrate that the performance of SM filtration can be directly impacted by variations in the feed composition applied in a priorly carried out filtration process. Our observations provide a basis for further investigations to mitigate fouling by aiming to form an optimized pre-fouling layer from other feed material or even permanent modifications of the membrane surface. An adapted pre-fouling system should focus on the exclusion of native CM and whey proteins from further fouling related to ion-induced interactions as well as size effects.

## Figures and Tables

**Table 1:** Concentrations of caseins and major whey proteins in skim milk (SM-0U), reconstituted MCC (MCC-0U), and MPI (MPI-0U) without Tgase treatment.

Feed Material	κ−Casein (mg mL^−1^)	α_s2_-Casein (mg mL^−1^)	α_s1_-Casein (mg mL^−1^)	β-Casein (mg mL^−1^)	α-Lac * (mg mL^−1^)	β-Lg ** B (mg mL^−1^)	β-Lg ** A (mg mL^−1^)
SM-0U	3.90 ± 0.15	4.90 ± 0.41	11.13 ± 0.38	11.92 ± 0.39	1.50 ± 0.16	1.36 ± 0.03	3.11 ± 0.10
MCC-0U	3.54 ± 0.09	3.64 ± 0.40	9.89 ± 0.35	10.97 ± 0.33	0.73 ± 0.11	0.58 ± 15	0.56 ± 0.02
MPI-0U	3.80 ± 0.04	3.71 ± 0.15	10.89 ± 0.17	11.86 ± 0.20	1.68 ± 0.02	1.99 ± 0.03	2.47 ± 0.03

* α-lac = α-lactalbumin; ** β-lg = β-lactoglobulin.

**Table 2:** Concentration of casein monomers derived from RP-HPLC of milk samples treated with different amounts of Tgase (0–3 U/g_prot_) and relative nativity compared to 0 U/g_prot_.

Tgase	κ-Casein		α_s2_-Casein		α_s1_-Casein		β-Casein	
Addition (U/g_prot_)	c (mg/mL)	Nativity (c_xU_/c_0U_)	c (mg/mL)	Nativity (c_xU_/c_0U_)	c (mg/mL)	Nativity (c_xU_/c_0U_)	c (mg/mL)	Nativity (c_xU_/c_0U_)
0	3.94	1.00	5.36	1.00	11.4	1.00	11.91	1.00
1	2.72	0.69	4.84	0.90	10.27	0.90	9.48	0.80
2	1.61	0.41	4	0.75	8.57	0.75	7.49	0.63
3	1.24	0.31	3.85	0.72	8.66	0.76	7.12	0.60

**Table 3:** Deposit resistance (R_D_), deposited mass (m_D_), and dry matter (DM) obtained after dead-end filtrations of differently pretreated SM at 0.5 and 3.0 bar ΔpTM.

Tgase Addition	R _D, 0.5 bar_	R _D, 3.0 bar_	m _D, 0.5 bar_	m _D, 3.0 bar_	DM _D, 0.5 bar_	DM _D, 3.0 bar_
(U/gprot)	(10^18^ m^−2^)	(10^18^ m^−2^)	(g)	(g)	(g)	(g)
0	6.95 ± 0.23	29.15 ± 1,54	0.219 ± 0.040	0.242 ± 0.019	0.083 ± 0.010	0.090 ± 0.005
1	7.39 ± 0.02	25.05 ± 0.25	0.243 ± 0.010	0.244 ± 0.001	0.094 ± 0.002	0.086 ± 0.14
2	6.15 ± 0.13	23.06 ± 1.42	0.228 ± 0.060	0.234 ± 0.018	0.081 ± 0.015	0.087 ± 0.02
3	6.02 ± 0.29	22.26 ± 2.41	0.256 ± 0.037	0.254 ± 0.026	0.094 ± 0.002	0.094 ± 0.01

## Data Availability

The original contributions presented in this study are included in the article. Further inquiries can be directed to the corresponding author.

## Abbreviations

The following abbreviations are used in this manuscript:

MF	Microfiltration
SM	Skim milk
MCC	Micellar casein concentrate
MPI	Milk protein isolate
R_D_	Deposit resistance
Δp_TM_	Transmembrane pressure difference
J	Flux
Tgase	Transglutaminase
DF	Diafiltration

## References

[1] De Boer R. (2014). From Milk By-Products to Milk Ingredients: Upgrading the Cycle.

[2] Pierre A., Fauquant J., Le Graet Y., Piot M., Maubois J.L. (1992). Préparation de phosphocaséinate natif par microfiltration sur membrane. Lait.

[3] Sisay E.J., Al-Tayawi A.N., László Z., Kertész S. (2023). Recent Advances in Organic Fouling Control and Mitigation Strategies in Membrane Separation Processes: A Review. Sustainability.

[4] Vetier C., Bennasar M., de la Fuente B.T. (1988). Study of the fouling of a mineral microfiltration membrane using scanning electron microscopy and physicochemical analyses in the processing of milk. J. Dairy Res..

[5] Qu P., Gésan-Guiziou G., Bouchoux A. (2012). Dead-end filtration of sponge-like colloids: The case of casein micelle. J. Membr. Sci..

[6] Huppertz T., Gazi I., Luyten H., Nieuwenhuijse H., Alting A., Schokker E. (2017). Hydration of casein micelles and caseinates: Implications for casein micelle structure. Int. Dairy J..

[7] Dalgleish D.G., Corredig M. (2012). The structure of the casein micelle of milk and its changes during processing. Annu. Rev. Food Sci. Technol..

[8] Trejo R., Dokland T., Jurat-Fuentes J., Harte F. (2011). Cryo-transmission electron tomography of native casein micelles from bovine milk. J. Dairy Sci..

[9] Reitmaier M., Bachmann I., Heidebrecht H.-J., Kulozik U. (2021). Effect of changes in ionic composition induced by different diafiltration media on deposited layer properties and separation efficiency in milk protein fractionation by microfiltration. Int. Dairy J..

[10] Adams M.C., Barbano D.M. (2013). Serum protein removal from skim milk with a 3-stage, 3× ceramic Isoflux membrane process at 50 °C. J. Dairy Sci..

[11] Adams M.C., Hurt E.E., Barbano D.M. (2015). Effect of soluble calcium and lactose on limiting flux and serum protein removal during skim milk microfiltration. J. Dairy Sci..

[12] Schorsch C., Carrie H., Clark A., Norton I. (2000). Cross-linking casein micelles by a microbial transglutaminase conditions for formation of transglutaminase-induced gels. Int. Dairy J..

[13] Huppertz T., de Kruif C.G. (2008). Structure and stability of nanogel particles prepared by internal cross-linking of casein micelles. Int. Dairy J..

[14] Akal H.C., Koçak C., Özer H.B., Budak S.O., Akal H.C. (2018). Transglutaminase Applications in Dairy Technology. Microbial Cultures and Enzymes in Dairy Technology.

[15] Smiddy M.A., Martin J.-E., Kelly A.L., de Kruif C.G., Huppertz T. (2006). Stability of Casein Micelles Cross-Linked by Transglutaminase. J. Dairy Sci..

[16] Mounsey J.S., O’Kennedy B.T., Kelly P.M. (2005). Influence of transglutaminase treatment on properties of micellar casein and products made therefrom. Lait.

[17] Puri R., Bot F., Singh U., O’Mahony J.A. (2021). Influence of Transglutaminase Crosslinking on Casein Protein Fractionation during Low Temperature Microfiltration. Foods.

[18] Vasbinder A.J., Rollema H.S., Bot A., de Kruif C.G. (2003). Gelation Mechanism of Milk as Influenced by Temperature and pH.; Studied by the Use of Transglutaminase Cross-Linked Casein Micelles. J. Dairy Sci..

[19] Gebhardt R., Steinhauer T., Meyer P., Sterr J., Perlich J., Kulozik U. (2012). Structural changes of deposited casein micelles induced by membrane filtration. Faraday Discuss..

[20] Schiffer S., Hartinger M., Matyssek A., Kulozik U. (2020). On the reversibility of deposit formation in low temperature milk microfiltration with ceramic membranes depending on mode of adjustment of transmembrane pressure and wall shear stress. Sep. Purif. Technol..

[21] De Kort E. (2015). Stabilized Micellar Casein and Compositions.

[22] Hinz K., Huppertz T., Kulozik U., Kelly A.L. (2007). Influence of enzymatic cross-linking on milk fat globules and emulsifying properties of milk proteins. Int. Dairy J..

[23] Gauche C., Vieira J.T., Ogliari P.J., Bordignon-Luiz M.T. (2008). Crosslinking of milk whey proteins by transglutaminase. Process Biochem..

[24] Salunke P., Metzger L.E. (2022). Impact of transglutaminase treatment given to the skim milk before or after microfiltration on the functionality of micellar casein concentrate used in process cheese product and comparison with rennet casein. Int. Dairy J..

[25] Chen L., Li Y., Han J., Yuan D., Lu Z., Zhang L. (2018). Influence of transglutaminase-induced modification of milk protein concentrate (MPC) on yoghurt texture. Int. Dairy J..

[26] Anantharaman A., Chun Y., Hua T., Chew J.W., Wang R. (2020). Pre-deposited dynamic membrane filtration—A review. Water Res..

[27] Li X.-Z., Hess H., Höflinger W. (2003). Influence of operating parameters on precoat layers built up under crossflow condition. Sep. Purif. Technol..

[28] Obermeyer H.D., Kulozik U., Kessler H.G. (1993). Controlled deposit formation to influence the retention of solutes in reverse osmosis and ultrafiltration. Desalination.

[29] Dumpler J., Kieferle I., Wohlschläger H., Kulozik U. (2017). Milk ultrafiltrate analysis by ion chromatography and calcium activity for SMUF preparation for different scientific purposes and prediction of its supersaturation. Int. Dairy J..

[30] Warncke M., Kulozik U. (2020). Impact of temperature and high pressure homogenization on the solubility and rheological behavior of reconstituted dairy powders of different composition. Powder Technol..

[31] Dumpler J., Wohlschläger H., Kulozik U. (2016). Dissociation and coagulation of caseins and whey proteins in concentrated skim milk heated by direct steam injection. Dairy Sci. Technol..

[32] Reiter M., Reitmaier M., Haslbeck A., Kulozik U. (2023). Acid gelation functionality of casein micelles in concentrated state: Influence of calcium supplementation or chelation combined with enzymatic stabilization. Food Hydrocoll..

[33] Li Y., Joyner H.S., Carter B.G., Drake M.A. (2018). Effects of fat content, pasteurization method, homogenization pressure, and storage time on the mechanical and sensory properties of bovine milk. J. Dairy Sci..

[34] Manji B., Kakuda Y. (1987). Determination of Whey Protein Denaturation in Heat-Processed Milks: Comparison of Three Methods. J. Dairy Sci..

[35] Svanborg S., Johansen A.-G., Abrahamsen R.K., Skeie S.B. (2014). Initial pasteurisation effects on the protein fractionation of skimmed milk by microfiltration. Int. Dairy J..

[36] Granger-Delacroix M., Leconte N., Garnier-Lambrouin F., Le Goff F., van Audenhaege M., Gésan-Guiziou G. (2020). Transmembrane Pressure and Recovery of Serum Proteins During Microfiltration of Skimmed Milk Subjected to Different Storage and Treatment Conditions. Foods.

[37] Steinhauer T., Lonfat J., Hager I., Gebhardt R., Kulozik U. (2015). Effect of pH, transmembrane pressure and whey proteins on the properties of casein micelle deposit layers. J. Membr. Sci..

[38] Lipnizki F., Boelsmand J., Madsen R.F. (2002). Concepts of industrial-scale diafiltration systems. Desalination.

[39] Nyambura H.L., Janssen A.E., van der Padt A., Boom R.M. (2025). A geometric model to predict protein retentions during skim milk microfiltration. J. Membr. Sci..

[40] Doudiès F., Loginov M., Hengl N., Karrouch M., Leconte N., Garnier-Lambrouin F., Pérez J., Pignon F., Gésan-Guiziou G. (2021). Build-up and relaxation of membrane fouling deposits produced during crossflow ultrafiltration of casein micelle dispersions at 12 °C and 42 °C probed by in situ SAXS. J. Membr. Sci..

[41] Ribadeau-Dumas B., Grappin R. (1989). Milk protein analysis: Review. Lait.

[42] (2002). Dried Milk—Assessment of Heat Treatment Intensity—Method Using High Performance Liquid Chromatography.

[43] Mazuknaite I., Guyot C., Leskauskaite D., Kulozik U. (2013). Influence of transglutaminase on the physical and chemical properties of acid milk gel and cottage type cheese. J. Food Agric. Environ..

[44] Guyot C., Kulozik U. (2011). Effect of transglutaminase-treated milk powders on the properties of skim milk yoghurt. Int. Dairy J..

[45] Lauber S., Henle T., Klostermeyer H. (2000). Relationship between the crosslinking of caseins by transglutaminase and the gel strength of yoghurt. Eur. Food Res. Technol..

[46] Creamer L.K., Berry G.P., Mills O.E. (1977). A study of the dissociation of beta -casein from the bovine casein micelle at low temperature. New Zealand J. Dairy Sci. Technol..

[47] Velazquez-Dominguez A., Hiolle M., Abdallah M., Delaplace G., Peixoto P.P. (2023). Transglutaminase cross-linking on dairy proteins: Functionalities, patents, and commercial uses. Int. Dairy J..

[48] Salunke P., Metzger L.E. (2023). Functional properties of milk protein concentrate and micellar casein concentrate as affected by transglutaminase treatment. Food Hydrocoll..

[49] Heidebrecht H.-J., Kulozik U. (2019). Fractionation of casein micelles and minor proteins by microfiltration in diafiltration mode. Study of the transmission and yield of the immunoglobulins IgG, IgA and IgM. Int. Dairy J..

[50] Raak N., Corredig M. (2022). Kinetic aspects of casein micelle cross-linking by transglutaminase at different volume fractions. Food Hydrocoll..

[51] Heidebrecht H.-J., Kainz B., Schopf R., Godl K., Karcier Z., Kulozik U., Förster B. (2018). Isolation of biofunctional bovine immunoglobulin G from milk- and colostral whey with mixed-mode chromatography at lab and pilot scale. J. Chromatogr. A.

[52] Weinberger M.E., Andlinger D.J., Kulozik U. (2021). A novel approach for characterisation of stabilising bonds in milk protein deposit layers on microfiltration membranes. Int. Dairy J..

[53] Hiller B., Lorenzen P.C. (2008). Surface hydrophobicity of physicochemically and enzymatically treated milk proteins in relation to techno-functional properties. J. Agric. Food Chem..

[54] Hartinger M., Heidebrecht H.-J., Schiffer S., Dumpler J., Kulozik U. (2019). Milk Protein Fractionation by Means of Spiral-Wound Microfiltration Membranes: Effect of the Pressure Adjustment Mode and Temperature on Flux and Protein Permeation. Foods.

[55] Ng K.S., Haribabu M., Harvie D.J., Dunstan D.E., Martin G.J. (2017). Mechanisms of flux decline in skim milk ultrafiltration: A review. J. Membr. Sci..

[56] Doudiès F., Arsène A.-S., Garnier-Lambrouin F., Famelart M.-H., Bouchoux A., Pignon F., Gésan-Guiziou G. (2019). Major Role of Voluminosity in the Compressibility and Sol-Gel Transition of Casein Micelle Dispersions Concentrated at 7 °C and 20 °C. Foods.

[57] Reitmaier M., Barbosa B., Sigler S., Heidebrecht H.-J., Kulozik U. (2020). Impact of different aqueous phases on casein micelles: Kinetics of physicochemical changes under variation of water hardness and diafiltration conditions. Int. Dairy J..

[58] Lu Y., McMahon D.J., Vollmer A.H. (2016). Investigating cold gelation properties of recombined highly concentrated micellar casein concentrate and cream for use in cheese making. J. Dairy Sci..

[59] Subhir S., McSweeney P.L., Murphy E.G., Tobin J.T. (2023). The impact of temperature on flux, protein transmission and energy usage during microfiltration of skim milk using polymeric membranes. Int. Dairy J..

[60] Gésan-Guiziou G., Boyaval E., Daufin G. (1999). Critical stability conditions in cross-flow microfiltration of skimmed milk: Transition to irreversible deposition. J. Membr. Sci..

[61] Thomar P., Nicolai T. (2016). Heat-induced gelation of casein micelles in aqueous suspensions at different pH. Colloids Surf. B Biointerfaces.

[62] Dunn M., Barbano D.M., Drake M. (2021). Viscosity changes and gel formation during storage of liquid micellar casein concentrates. J. Dairy Sci..

[63] Balakrishnan G., Silva J.V.C., Nicolai T., Chassenieux C., Bovay C., Buczkowski J., Schmitt C. (2018). Specific effect of calcium ions on thermal gelation of aqueous micellar casein suspensions. Colloids Surf. B Biointerfaces.

[64] Bryant C.M., McClements D.J. (2000). Influence of NaCl and CaCl_2_ on Cold-Set Gelation of Heat-denatured Whey Protein. J. Food Sci..

[65] Kharlamova A., Nicolai T., Chassenieux C. (2018). Mixtures of sodium caseinate and whey protein aggregates: Viscosity and acid- or salt-induced gelation. Int. Dairy J..

[66] Alting A.C., Hamer R.J., de Kruif C.G., Paques M., Visschers R.W. (2003). Number of thiol groups rather than the size of the aggregates determines the hardness of cold set whey protein gels. Food Hydrocoll..

[67] Jimenez-Lopez A., Leconte N., Garnier-Lambrouin F., Bouchoux A., Rousseau F., Gésan-Guiziou G. (2011). Ionic strength dependence of skimmed milk microfiltration: Relations between filtration performance, deposit layer characteristics and colloidal properties of casein micelles. J. Membr. Sci..

[68] Fu L.F., Dempsey B.A. (1998). Modeling the effect of particle size and charge on the structure of the filter cake in ultrafiltration. J. Membr. Sci..

